# RAVL1, an upstream component of brassinosteroid signalling and biosynthesis, regulates ethylene signalling via activation of *EIL1* in rice

**DOI:** 10.1111/pbi.12925

**Published:** 2018-04-24

**Authors:** Xiao Feng Zhu, De Peng Yuan, Chong Zhang, Tian Ya Li, Yuan Hu Xuan

**Affiliations:** ^1^ College of Plant Protection Shenyang Agricultural University Shenyang Liaoning China

**Keywords:** brassinosteroids, ethylene, RAVL1, EIL1, rice

Brassinosteroids (BRs) are steroid hormones that regulate several aspects of plant growth and development. Their signal is activated by the binding of BRs to the receptor brassinosteroid‐insensitive 1 (BRI1), which activates the transcription factors brassinazole‐resistant 1 (BZR1) and BRI1‐EMS‐SUPPRESSOR 1 (BES1) that regulate target gene expression (Kim and Wang, 2010). More recently, the crosstalk between BRs and other hormone signalling factors has been extensively investigated, and it has been suggested that BR signalling is highly integrated with other signals to regulate plant growth and development (Wang *et al*., 2012). We previously identified Related to ABI3/VP1RAV‐Like 1 (RAVL1), a transcriptional activator that modulates BR homeostasis by activating *BRI1* and biosynthetic genes in rice (Je *et al*., 2010). However, RAVL1 is not modulated by BRs, suggesting a potential association between BRs and other hormones via RAVL1 in rice.

To investigate whether RAVL1 is involved in the crosstalk circuits between BRs and other hormones, the hormone‐dependent expression of *RAVL1* was analysed. Seven‐day‐old wild‐type plants were subjected to various treatments using 2,4‐dichlorophenoxyacetic acid (2,4‐D), epibrassinolide (epi‐BL), abscisic acid (ABA), gibberellic acid (GA) and 1‐aminocyclopropane‐1‐carboxylic acid (ACC), and the *RAVL1* level was monitored by quantitative real‐time PCR (qPCR). The results indicated that 2,4‐D (a synthetic auxin) treatment induced while ACC application suppressed the *RAVL1* expression level, but the other hormones examined did not alter the *RAVL1* level (Figure 1a). Analysis of the *RAVL1* promoter sequences revealed an auxin‐responsive element located within 1 kb of these promoter sequences (data not shown), suggesting that auxin response factors (ARFs) might be involved in auxin induction of *RAVL1*. However, further analyses are required to determine whether ARFs bind to *RAVL1* promoter sequences. To further investigate the effect of ethylene signalling on *RAVL1*,* RAVL1* expression levels were examined in an *ethylene‐insensitive 2* (*EIN2*) mutant, *ethylene‐insensitive 3‐like 1* (*EIL1*) *RNAi* (*eil1 Ri*), *ethylene response sensor 1* (*ERS1*) mutant and *EIL1* overexpressor (*EIL1 OX*). The data indicate that *RAVL1* was slightly higher in *ein2* and *eil1 Ri*, while lower in *ers1* and *EIL1 OX* compared with wild‐type plants (Figure 1b). As *RAVL1* levels were suppressed by ACC, ACC‐dependent root growth was analysed in wild‐type, *RAVL1* mutants (*ravl1‐1* and *ravl1‐2*) and *RAVL1* overexpression lines (*RAVL1 OX* and *RAVL1‐GFP OX*). Primary root growth was inhibited by ACC treatment in wild‐type plant, and the inhibition rate was lower in *RAVL1* mutants and higher in *RAVL1* overexpression lines than in wild‐type plants (Figure 1c,d), indicatingthat RAVL1 positively contribute to the ACC‐mediated root growth suppression.

**Figure 1:**
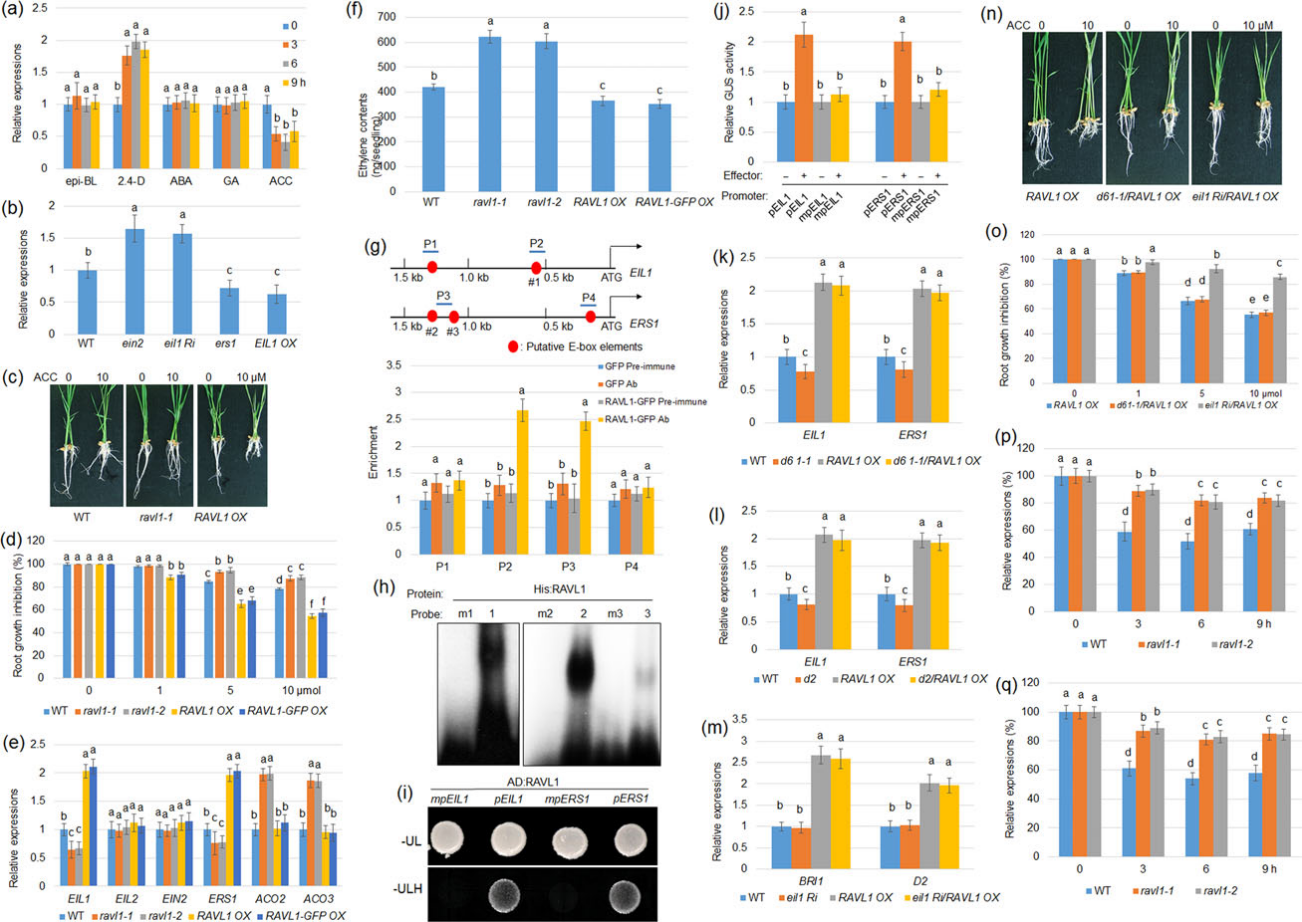
RAVL1 directly activates *EIL1* and *ERS1* regardless of brassinosteroids (BRs) signalling. (a) The expression patterns of *Related to ABI3/VP1RAV‐Like 1* (*RAVL1*) were monitored by quantitative real‐time PCR (qPCR) performed on 7‐day‐old wild‐type (WT) seedlings treated with 1 μm of epibrassinolide (epi‐BL), 2,4‐dichlorophenoxyacetic acid (2,4‐D), abscisic acid (ABA), 1‐aminocyclopropane‐1‐carboxylic acid (ACC) and gibberellic acid (GA). *RAVL1* expression patterns were evaluated after 0, 3, 6 and 9 h of treatment. Error bars represent ± standard error (SE) of three repeated experiments (*n* = 3). (b) *RAVL1* level was examined in 7‐day‐old WT,* ein2*,* eil1 Ri*,* ers1* and *EIL1 OX* plants. *Ubiquitin* was used as a control to normalize data. Error bars represent ± SE (*n* = 3). (c) Seven‐day‐old WT,* ravl1‐1* mutant and *RAVL1* overexpression (OX) plants analysed for 10 μm ACC‐dependent root growth were photographed. (d) ACC‐dependent root growth of wild‐type, *ravl1‐1*,* ravl1‐2*,*RAVL1 OX* and *RAVL1‐green fluorescence protein (GFP) OX* was calculated. Error bars indicate ± SE (*n* = 10). (e) Expression of *EIL1*,*EIL2*,*EIN2*,*ERS1, ACO2* and *ACO3* was examined in WT,* ravl1‐1*,* ravl1‐2*,*RAVL1 OX* and *RAVL1‐GFP OX* plants. Error bars indicate ± SE (*n* = 3). (f) Ethylene contents were measured from 7‐day‐old WT,* ravl1‐1*,* ravl1‐2*,*RAVL1 OX* and *RAVL1‐GFP OX* plants. Error bars indicate ± SE (*n* = 30). (g) Schematic diagram indicating E‐box location (red circle) within 1.5 kb *EIL1* and *ERS1* promoters, and probes (P) used for chromatin immunoprecipitation (ChIP) assays. Numbers (1–3) below E‐box elements indicate probes used in the electrophoretic mobility shift assay (EMSA). Relative ratios of immunoprecipitated DNA to input DNA were determined by qPCR. Input DNA was used to normalize the data. Pre‐immune serum: IgG, Ab: GFP antibody. Error bars indicate ± SE (*n* = 3). (h) EMSA was performed to evaluate RAVL1 affinities to each of the three E‐box elements (numbers 1–3) and mutated probes (m1–m3). (i) A yeast one‐hybrid assay was conducted to analyse the RAVL1 activation within 1.5 kb of *EIL1* and *ERS1* promoters. Yeast cells harbouring either *AD‐RAVL1* and *pEIL1‐His*,*AD‐RAVL1* and *pERS1‐His*, or E‐box‐mutated promoters *mpEIL1‐His* and *mpERS1‐His*, respectively, were grown on SD media lacking leucine (Leu) and uracil (Ura) or Leu, Ura and histone (His). (j) A transient expression assay was performed by co‐transfection with *p35S:RAVL1* and each of the vectors expressing the beta‐glucuronidase gene (*GUS*) under the control of native and E‐box‐mutated *EIL1* or *ERS1* promoters in protoplast cells. The luciferase gene driven by the 35S promoter was used as an internal control to normalize *GUS* expression. Error bars indicate ± SE (*n* = 6). *EIL1* and *ERS1* expression levels were tested in *d61‐1* (a BRI1 mutant) and *RAVL1 OX* genetic combinations (k) or *d2* and *RAVL1 OX* genetic combination plants (l). Error bars indicate ± SE (*n* = 3). (m) *BRI1* and *D2* levels were monitored in *eil1* and *RAVL1 OX* genetic combinations. Error bars indicate ± SE (*n* = 3). (n) *RAVL1 OX*,* d61‐1/RAVL1 OX* and *eil1 Ri*/*RAVL1 OX* 7‐day‐old plants were analysed for 1‐aminocyclopropane‐1‐carboxylic acid (ACC)‐dependent root growth (10 μm ACC‐treated and control plants were examined). (o) ACC‐dependent root growth inhibition rates of *RAVL1 OX*,* d61‐1/RAVL1 OX* and *eil1 Ri*/*RAVL1 OX* were calculated. Data are presented as means ± SE (*n* = 10). The ACC‐dependent expression patterns of *BRI1* (p) and *D2* (q) were evaluated after 0, 3, 6 and 9 h of treatment. Error bars indicate ± SE (*n* = 3). The relative expression rate was calculated against 0 h time point. All experiments were repeated at least three times. For all experiments, one‐way analysis of variance (ANOVA) was conducted, followed by Bonferroni's multiple comparison tests. Significant differences at *P *<* *0.05 level are indicated by different lowercase letters.

The expression of *EIL1*,* EIL2*,* EIN2*,* ERS1*,* ACC oxidase 2* (*ACO2*) and *ACO3* was analysed in *RAVL1* mutants and *RAVL1* overexpression lines by qPCR. *EIL1* and *ERS1* levels were lower in mutants and higher in overexpression lines than in wild‐type plants. *ACO2* and *ACO3* levels were higher in mutants than in wild‐type and overexpression lines, while *EIN2* and *EIL2* levels did not change among the tested lines (Figure 1e). As the expression of *ACO2* and *ACO3* (two ethylene biosynthesis enzymes) was altered in *ravl1‐1* mutants, ethylene content was measured. The results demonstrated that *ravl1* mutants accumulated more ethylene, while *RAVL1* overexpressors contained less ethylene compared with wild‐type plants (Figure 1f). As *EIL1* and *ERS1* are key ethylene signalling genes positively regulated by RAVL1, their promoter sequences were analysed to identify the presence of E‐box elements in the RAVL1‐binding region. Two and three putative E‐box elements were located within 1.5 kb of *EIL1* and *ERS1* promoters, respectively (Figure 1g). To test the binding affinity of RAVL1 to the E‐box elements, a chromatin immunoprecipitation assay was performed using 35S:GFP and 35S:RAVL1:GFP transgenic plant calli. A pre‐immune serum was used as the control for the GFP antibody to immunoprecipitate DNA. RAVL1 bound to the P2 and P3 regions of *EIL1* and *ERS1* promoters, respectively (Figure 1g). We further performed an electrophoretic mobility shift assay to determine which E‐box elements were responsible for the binding affinity to RAVL1, using six probes. We found that RAVL1 bound to E‐boxes in positions ‘1’, ‘2’ and ‘3’, but failed to bind their mutated probes m1, m2 and m3 (Figure 1h). The results of this binding assay were confirmed using a yeast one‐hybrid assay, which indicated that RAVL1 is only able to activate 1.5‐kb *EIL1* and *ERS1* promoters (*pEIL1* and *pERS1*) if the E‐box promoters are not mutated at P2 and P3 regions of *EIL1* and *ERS1* promoters, respectively (*mpEIL1* and *mpERS1*). In these mutated promoters, E‐box element sequences CANNTG were substituted by the sequence TTTTTT (Figure 1i). To verify whether these *cis*‐elements were responsible for the transcriptional activation of *EIL1* and *ERS1* promoters by RAVL1 *in vivo*, we performed transient expression assays using the protoplast system. Protoplast cells were co‐transformed with the 35S:RAVL1 plasmid and a vector expressing the beta‐glucuronidase gene (*GUS*) under the control of *pEIL1* and *pERS1* or *mpEIL1* and *mpERS1*. Using 35S:Luciferase (LUC) as an internal control to normalize the transformation efficiency in each assay, protoplasts expressing RAVL1 had approximately twice the levels of activated *pEIL1* and *pERS1*, but RAVL1 was unable to activate *mpEIL1* and *mpERS1* (Figure 1j). These results indicate that RAVL1 directly activates *EIL1* and *ERS1* via promoter binding. More interestingly, RAVL1 is able to directly activate *BRI1* and *ERS1*, and directly regulates other key genes in BR biosynthesis (*D2* and *D11*; Je *et al*., 2010) and ethylene signalling (*EIL1*). *RAVL1* mutants are insensitive to BRs and ethylene, two important phytohormones, while *RAVL1* overexpression lines are sensitive to both, which is in agreement with existing molecular data, because RAVL1 activate BR‐ and ethylene‐related genes. ERS1 is a negative regulator of ethylene signalling and function at the upstream of *EIL1* (Yang *et al*., 2015a,b), and its mutant is sensitive to ethylene in rice (Ma *et al*., 2014). The positive regulation of RAVL1 on ethylene response might be caused by the activation of *EIL1*, which may cover the activation of *ERS1* by RAVL1.

RAVL1 directly activates *BRI1* and biosynthetic genes to maintain BR homeostasis (Je *et al*., 2010). We further examined whether RAVL1 activation of *EIL1* and *ERS1* depends on BR signalling using genetic combinations between *RAVL1 OX* and *d61‐1*, a mutant of *BRI1*, or between *RAVL1 OX* and *d2*, a BR biosynthetic gene mutant, and qPCR was performed. *EIL1* and *ERS1* expression levels were slightly lower in *d61*‐*1* and higher in *RAVL1 OX* plants compared with wild‐type plants. However, the mutation of *BRI1* in the *RAVL1 OX* background (*d61‐1/RAVL1 OX*) did not affect *EIL1* and *ERS1* activation by RAVL1 (Figure 1k). Similarly, *EIL1* and *ERS1* expression levels were lower in *d2* compared with wild‐type plants; however, *D2*‐mutated plants showed no difference in the activation of *EIL1* and *ERS1* by RAVL1 (Figure 1l), suggesting that RAVL1 might activate *EIL1* and *ERS1* in BR signalling‐independent manner. Activation of *BRI1* and *D2* by RAVL1 was examined in *eil1 Ri* and *RAVL1 OX* genetic combinations. The data showed that the knock‐down of *EIL1* did not affect the activation of *BRI1* and *D2* by RAVL1 (Figure 1m), indicating that RAVL1 might be also independent of ethylene signalling to activate *BRI1* and *D2*. Ethylene sensitivity of *RAVL1 OX*,* d61*‐*1/RAVL1 OX* and *eil1 Ri/RAVL1 OX* was further examined, and the results indicated that *RAVL1 OX* and *d61‐1/RAVL1 OX* exhibited similar responses to exogenously applied ethylene, while *eil1 Ri/RAVL1 OX* reduced its sensitivity to ethylene (Figure 1n,o). These results suggest that RAVL1 is required in the ACC‐induced root growth suppression, which independents of BR signalling but requires ethylene signalling. However, *EIL1* and *ERS1* levels were slightly lower in *d61‐1* and *d2* mutants, suggesting that BR signalling might be upstream of ethylene signalling. In addition, treatment of an ethylene precursor ACC suppresses *RALV1* expression, and the *RAVL1* level was reduced in *ers1* and *EIL1 OX* plants. Moreover, RAVL1 positively contributes to the ethylene signalling, implying that feedback regulation may occur between *RAVL1* and ethylene signalling. Similarly, the suppression of *RAVL1* by ethylene might be a result of the activation of ethylene signalling.

To analyse whether ethylene also influences BR‐related genes via RAVL1, ACC‐mediated expressions of *BRI1* and *D2* were further examined in 7‐day‐old wild‐type and *ravl1* mutants. As the *BRI1* and *D2* levels were lower in *ravl1* mutants than in wild‐type plants (Je *et al*., 2010), ACC‐mediated expressions of *BRI1* and *D2* were calculated as ratio to untreated control. The RT‐qPCR results indicated that ACC application suppressed *BR1I* and *D2* expression, but the suppression was partially inhibited in *ravl1* mutants (Figure 1p,q), suggesting that *RAVL1* is involved in the ethylene‐mediated BR signalling gene suppression. Further molecular experiments are necessary to isolate the ethylene signalling factors that inhibit *RAVL1* levels. Overall, our findings provided useful insight into the BR and ethylene crosstalk in rice. More importantly, the data revealed that RAVL1 activates both BR and ethylene signalling in rice.

## Conflict of interest

The authors declare no conflict of interest.
